# Relationship between pulp-tooth volume ratios and chronological age in different anterior teeth on CBCT

**DOI:** 10.4317/jced.53654

**Published:** 2017-05-01

**Authors:** Nima Biuki, Tahmineh Razi, Masoumeh Faramarzi

**Affiliations:** 1 Assistant Professor, Department of Oral and Maxillofacial Radiology, Faculty of Dentistry, Tabriz University of Medical Sciences, Tabriz, Iran; 2 Assistant Professor, Department of Oral and Maxillofacial Radiology, Faculty of Dentistry, Tabriz University of Medical Sciences, Tabriz, Iran; 3 Associated Professor, Department of Periodontics, Faculty of Dentistry, Tabriz University of Medical Sciences, Tabriz, Iran

## Abstract

**Background:**

The CBCT imaging technique exhibits proper accuracy to determine the internal anatomy of teeth. Therefore, this technique can use to estimate age by measuring the amount of decrease in the volume of the pulpal cavity of teeth. The aim of the present study was to evaluate the correlation between chronological age and pulp-to-tooth volume ratios in anterior teeth with the use of the CBCT technique and to determine a regression model to estimate human age.

**Material and Methods:**

In this present descriptive-analytical study, 122 subjects (46 males and 76 females), with an age range of 13-70 years, were evaluated. The MIMICS software program was used to determine the pulp-to-tooth volume ratios in 732 anterior teeth. Regression analysis was used to assess the correlation between age and pulp to tooth volume ratios.

**Results:**

In all the teeth evaluated, there was an inverse and significant correlation between age and the pulp-to-tooth volume ratios in males and females, with a stronger correlation in males. In addition, such a correlation was stronger in maxillary central incisors and canines. In the model in which the mean of ratios in anterior teeth was used to estimate age the correlation was stronger compared to that in single tooth.

**Conclusions:**

In general, the results of the present study showed that it is advisable to use the mean of all the ratios of anterior teeth in forensics to estimate age.

** Key words:**Age estimation , cone-beam CT, forensic dentistry, secondary dentin, pulp cavity.

## Introduction

It is obvious that it is very important to identify of living individuals and cadavers in forensics; in this context, an important aspect of identification is the estimation of the chronological age of individuals. One of the most important responsibilities of forensics is to determine the age of convicts, criminals, illegal immigrants and remains of human bodies after accidents (1). There are different techniques to determine age, depending on the age range of individuals. Age estimation is carried out up to 24 years of age based on eruption sequence and the developmental stages of teeth; however, estimation of age in adults after maturation of third molars is a matter of controversy (2-4).

To date several techniques have been explained to determine age in adult populations based on morphologic and structural changes in teeth. Many of these techniques are destructive methods and the teeth should be extracted to apply them and therefore they cannot be used in living individuals (5). Deposition of secondary dentin occurs throughout life on all the pulpal walls, resulting in a continuous decrease in the size of the pulpal cavity. Therefore, age estimation in adults can be carried out by measuring the amount of decrease in the size of the pulpal cavity of teeth (5,6). The amount of such a decrease in size has been determined in different adult populations with the use of conventional dental radiographs and different results have been achieved (7-10). However, conventional radiographs have various limitations, including superimposition and no equal magnification (11,12). To overcome such limitations use of three-dimensional imaging such as micro-CT with high resolution has been suggested. However, this technique can be applied only to extracted teeth due to the limited FOV (field of view) of the area involved.

In a preliminary study with the use of CBCT imaging technique, the pulp-to-tooth volume ratios were calculated in single-rooted teeth with the use of primitive custom-made software program and a formula was designed to estimate age with a determination coefficient of 29%. In addition, it was reported that it is possible to calculate pulp-to-tooth volume ratios in living individuals by three-dimensional evaluation of CBCT images (11).

A study by Star et al. (13) showed a poor correlation between the pulp-to-tooth volume ratios in mandibular and maxillary incisors, canines and premolars. In that study, only one single-rooted tooth was selected randomly in each subject. Therefore, further studies are necessary given advances in CBCT technology in order to determine the best method to estimate age.

In a recent study, the pulp-to-tooth volume ratios were evaluated in all the 4 canine teeth on 210 CT scan images. Based on the results, seven models were suggested using the least square weighted method with a high correlation coefficient to estimate the age of adult populations, among which the combined model for maxillary canines was more efficacious. In that study, only the canine teeth were evaluated, which does not appear to be adequate because these teeth might be missing in an individual. In addition, the study was carried out with the use of CT imaging technique which cannot provide dental images with high spatial resolution (14).

The aim of the present study was to evaluate the correlation between chronological age and pulp-to-tooth volume ratios in 6 anterior teeth in an Iranian population and also to determine a regression model for estimation of chronological age using CBCT radiographs with proper accuracy for legal medicine purposes.

## Material and Methods

In the present descriptive-analytical study, 122 subjects referring to the maxillofacial radiology department of Tabriz dentistry faculty, who required a CBCT examination as part of their routine examination and treatment planning, were enrolled on the study. All the subjects had at least 6 fully developed and intact teeth with closed apices, including maxillary and mandibular central and lateral incisors and canines. Due to bilateral symmetry of tooth internal anatomy, right or left teeth were selected in a random manner. All of the selected teeth had one root with single canal.

The subjects underwent clinical and radiographic examinations and those with dental caries, occlusal traumas and periodontal diseases in the above-mentioned teeth or those with a history of trauma or syndromes affecting the teeth were excluded. In addition, radiographic images with evidence of pathologic lesions in the soft and hard tissues surrounding the teeth, dental anomalies, internal or external resorption, pulp stones, diffuse pulpal sclerosis, teeth with restorative, endodontic, prosthetic and orthodontic treatments, and images with poor quality (including artifacts resulting from the patient movement, beam hardening etc.) were excluded.

All the images were taken with a NewTom VGi (QR srl, Verona, Italy) with a cone-beam shaped, a rotation of 360° and a flat-panel image detector with a pixel size of 0.150 mm and with F.O.V size of 12*8cm. The exposure conditions consisted of pulsed exposure at 110 KVP, variable current at 1-20 mA and a total scan time of 18 seconds.

**Figure 1:**
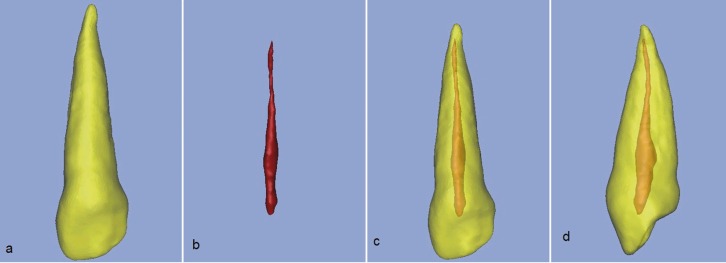
An upper left canine in axial view (a), The same view with segmented masks of pulp and tooth (b), The tooth in longitudinal view (c) and the same view with segmented masks (d).

**Figure 2:**
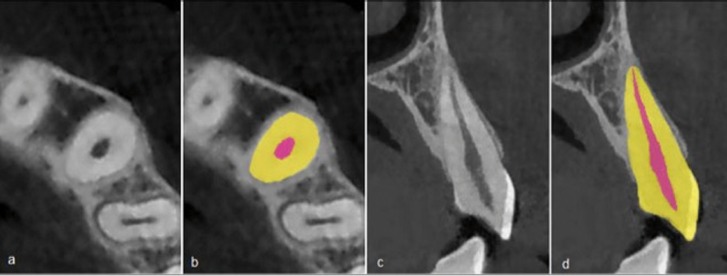
A 3D segmented upper left canine (a),3D segmented pulp of same tooth (b),Tooth & pulp of that tooth in anterior view and (c) and in lateral view (d).

The images were exported in the DICOM format using the NNT viewer software program (Version 2.21) and the MIMICS software program (Version 10.01) (Materialise N.V., Belgium) was used to carry out volumetric calculations. Each tooth in question was segmented in a 3-dimensional manner in the images provided using the above software program. To this end, first the image of each tooth was cropped in axial, coronal and sagittal dimensions. Then the minimum and maximum thresholds of grey level of each tooth were used to produce a mask. In the next stage the operator removed all images structures other than the tooth in question in all cross-section view at a thickness of 0.3 mm, which included the images of the cortical bone, lamina dura, the adjacent tooth etc. Then the dimensions of the defined mask were re-evaluated in the longitudinal sections (Fig. 1). Finally, the three-dimensional object was reconstructed and volumetric measurements were carried out in cubic millimeters using the software program. In addition, all the steps above were repeated for the pulp structures of all the teeth and their volumes were calculated by the same software program (Fig. 2). The pulp-to-tooth volume ratios were calculated for all the teeth. All the computer images were visualized in a dimlylit room on a 32-bit and 19-inch LCD monitor (PHILIPS, 190B) with a resolution of 1208*1024. First the pulp and the whole tooth volumes were calculated in all the 6 teeth under study on the radiographic images of 15 subjects by operator 1 and 2, who were adequately trained to be able to calculate volumes with the use of the software program. Then the intra-class correlation coefficient was calculated for the two observers (ICC=0.75) and the rest of the procedures were carried out by operator 1. Pearson correlation coefficient was used to evaluate the correlation between chronological age and the pulp to tooth volume ratios in terms of tooth type and gender. Then the simple linear regression model with the effect of age was considered as the dependent variable and the pulp to tooth volume ratio was considered as the predictive variable to determine the formula to estimate chronological age. In this regression model, the relationships between age and pulp-to-tooth volume ratios were compared between males and females and the teeth type under study. Data were analyzed with SPSS 17. Statistical significance was set at *P*≤0.05.

## Results

Of 122 subjects in the present study, 46 (37.7%) and 76 (62.3%) subjects were male and female, respectively. The mean ages of males and females were 39.82 (a range of 15.90-69.54) and 36.62 (a range of 13.60-61.85) years, respectively.  Table 1 shows the pulp volumes, tooth volumes and the pulp to tooth volume ratios of all the six teeth under study.

**Table 1:**
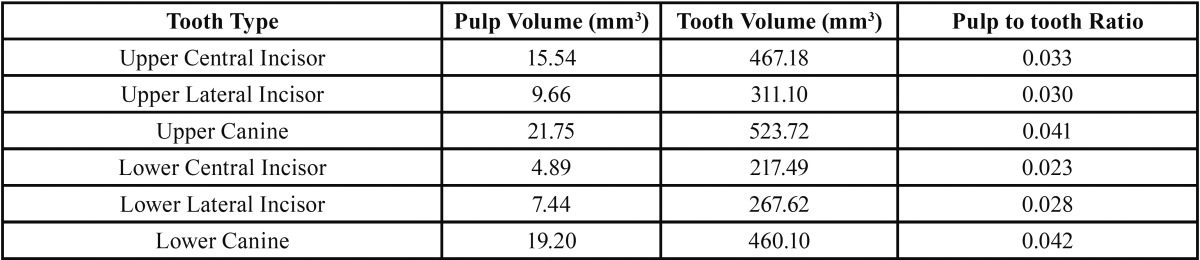
Mean pulp volumes, tooth volumes and pulp to tooth volume ratios of all the six tooth types.

**Figure 3:**
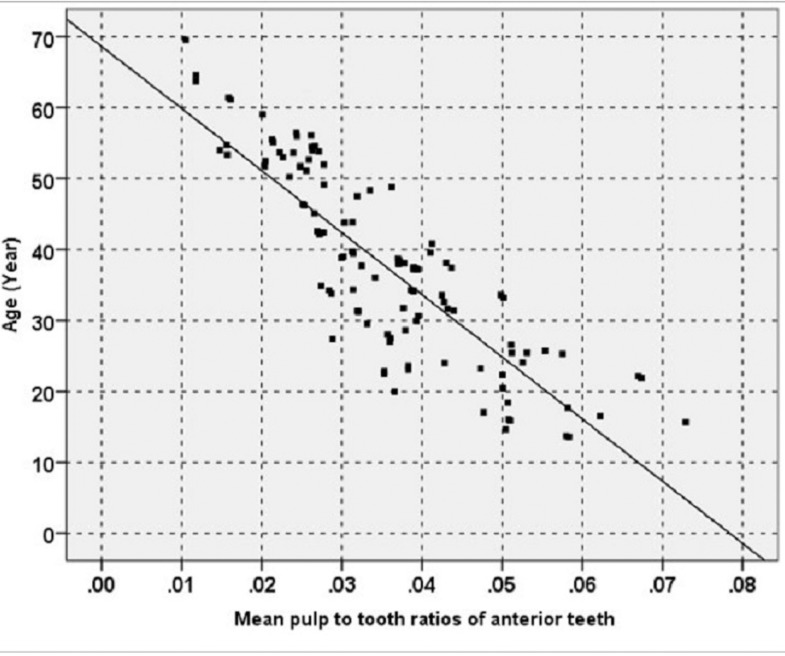
Scatter plot and estimated line represent a linear regression relation between mean pulp to tooth volume ratios of anterior teeth and age.

 Table 2 presents Pearson correlation coefficients between age and pulp to tooth volume ratios and also the standard errors of estimates separately for both sexes in the all six tooth types under study. In all the six tooth types in both males and females there was an inverse and significant correlation between the two variables mentioned above (*P*<0.001), with a stronger correlation in males compared to females. Scatter plot of mean pulp to tooth volume ratios of anterior teeth and age shows a linear regression relation (Fig. 3). The regression formula was separately calculated for each tooth type in both genders. Formula determined with the use of volume ratios in the mean ratios of the all anterior teeth, the maxillary central incisors and maxillary canines in both sexes respectively exhibited least error. These formulae are as follow:

**Table 2:**
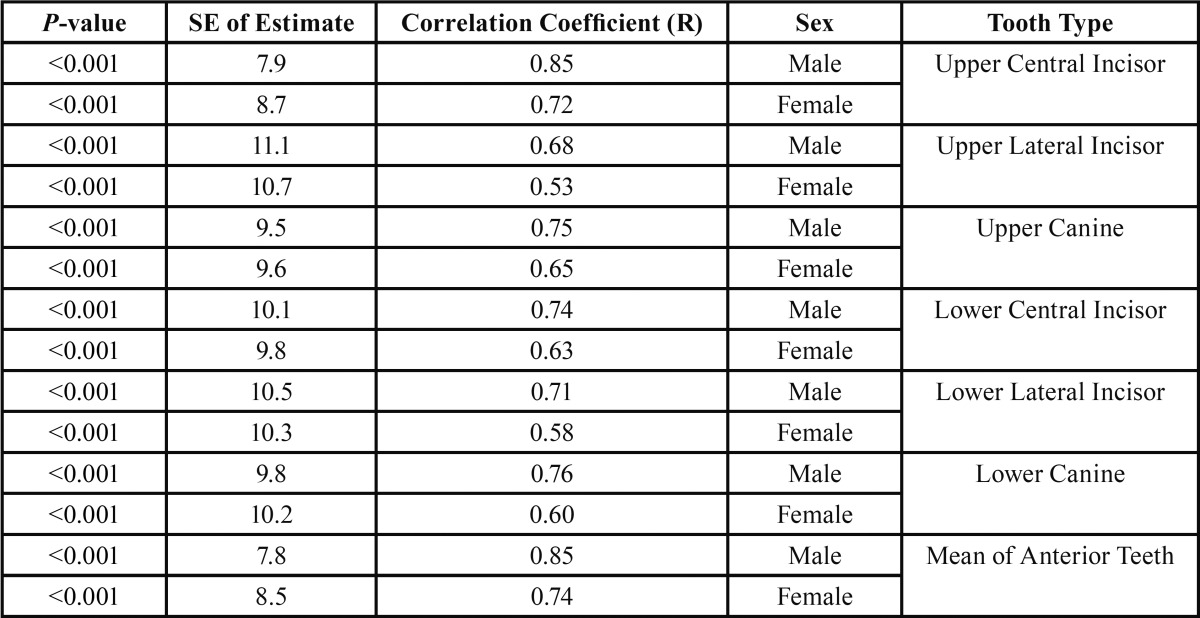
Spearman’s correlation coefficients between age and pulp to tooth volume ratios and the standard errors of estimates separately for both sexes.

Upper Central Incisor in male: Y= 77/66 -921 X

Upper Lateral Incisor in male: Y= 33/60 - 700 X

Upper Canine in male: Y= 2/71- 793X

Lower Central Incisor in male: Y89/53= -658 X

Lower Lateral Incisor in male: Y=49/58 730 -X

Lower Canine in male: Y=89/71 -795 X

Mean of Anterior Teeth in male: Y=95/72 - 1010X

Upper Central Incisor in female: Y=01/59 - 621X

Upper Lateral Incisor in female: Y=15/50 -427 X

Upper Canine in female: Y=23/62 - 603X

Lower Central Incisor in female: Y11/50= 556 -X

Lower Lateral Incisor in female: Y=9/52 553-X

Lower Canine in female: Y= 39/65- 567X

Mean of Anterior Teeth in female: Y= 39/65- 787X

## Discussion

Previous studies have shown that CBCT imaging technique has adequate accuracy for evaluation of the anatomy of the pulp cavity (15) and it can be used in living individuals in a non-aggressive technique by using a single scanning to collect useful data about the 3-dimensional structure of teeth (16). Therefore, determination of tooth volume with the use of the CBCT technique is an acceptable in vivo method, with an error rate of 4-7% (17). To this end we used the CBCT technique for 3-dimensional modeling of teeth, the pulp and determining their volumes.

Since it is not possible to select several teeth in one individual to determine a regression model to estimate age, the non-equal number of ratios calculated in one individual are not suitable and each ratios in one individual should be used separately to determine a regression model, in the present study 6 types of equal anterior teeth type were selected to evaluate and compare the predictive value for age in different teeth type (13).

Based on the results of the present study, the mean of pulp-to-tooth volume ratios in maxillary and mandibular canines was higher than that in other teeth, which is expected by taking account of the anatomy, morphology and the size of canines and the minor effect of irritating factors on secondary dentin formation (18).

The results of the present study, consistent with those of previous studies, showed an inverse correlation between the pulp-to-tooth volume ratios and age in all the teeth evaluated, i.e. there is a decrease in pulp volume with aging. Such a correlation was stronger in the maxillary central incisors and canines compared to other teeth, which might be explained by less internal anatomical variations in these teeth (18). In addition, it is easier and more accurate to separate the pulpal area in teeth with one root canal with longer dimensions using software techniques. Furthermore, the results of the present study showed that such a correlation was stronger in males compared to females, i.e. the pulp-to-tooth volume ratio is a more favorable variable in males compared to females to estimate age, indicating that gender affects the formula used to estimate age, consistent with the results reported by Tardivo *et al.* (14) in relation to canines. However, in a study by Star *et al.* (13), the correlation was stronger in females compared to males. Such a discrepancy in the results might be attributed to differences in the samples between these two studies and simultaneous evaluation of maxillary central and lateral incisors in the incisor group and maxillary and mandibular canines in the canine group in the study carried out by Star.

In a large number of studies canine teeth have been used to estimate age because these teeth have larger dimensions, bigger pulps and more stability compared to other one rooted teeth. For example, in a study by Tardivo in the seven models determined to estimate age, maxillary and mandibular canines were used, which yielded good results (14). However, a study in which only mandibular central incisors were used, the results were less valid (12). Tardivo *et al.* (14) reported that simultaneous use of two maxi-llary canines are better and stronger in estimating age compared to the use of four human canine teeth, indicating the higher capacity of maxillary canines, compared to mandibular canines, for estimating age that consistent with the results of the present study. These conclusions might be attributed to the larger size and less anatomic variations of these teeth.

## Conclusions

Of all the models determined to estimate age, the model in which the mean pulp-to-tooth volume ratios of six anterior teeth were used, exhibited higher accuracy in estimating age. If it is not possible to simultaneously use six anterior teeth, maxillary central incisors and canines might be used.
